# Population structure of a global agricultural invasive pest, *Bactrocera dorsalis* (Diptera: Tephritidae)

**DOI:** 10.1111/eva.12701

**Published:** 2018-09-28

**Authors:** Yu‐jia Qin, Matthew N. Krosch, Mark K. Schutze, Yue Zhang, Xiao‐xue Wang, Chandra S. Prabhakar, Agus Susanto, Alvin K. W. Hee, Sunday Ekesi, Kemo Badji, Mahfuza Khan, Jia‐jiao Wu, Qiao‐ling Wang, Ge Yan, Li‐huan Zhu, Zi‐hua Zhao, Li‐jun Liu, Anthony R. Clarke, Zhi‐hong Li

**Affiliations:** ^1^ Department of Entomology College of Plant Protection China Agricultural University Beijing China; ^2^ School of Earth, Environmental and Biological Sciences Queensland University of Technology (QUT) Brisbane Queensland Australia; ^3^ Department of Entomology Bihar Agricultural University Bhagalpur Bihar India; ^4^ Faculty of Agriculture Padjadjaran University Jatinangor Indonesia; ^5^ Department of Biology, Faculty of Science Universiti Putra Malaysia Selangor Malaysia; ^6^ International Centre of Insect Physiology and Ecology Nairobi Kenya; ^7^ Fruit Fly Control Project‐ECOWAS Responsable Composante Surveillance. Projet Lutte contre les Mouches des Fruits‐CEDEAO CRSA Bamako Mali; ^8^ Insect Biotechnology Division Institute of Food and Radiation Biology Atomic Energy Research Establishment Savar, Dhaka Bangladesh; ^9^ Guangdong Inspection and Quarantine Technology Center Guangzhou China

**Keywords:** *Bactrocera dorsalis*, geometric morphometrics, microsatellites, mitochondrial genes, population structure

## Abstract

*Bactrocera dorsalis,* the Oriental fruit fly, is one of the world's most destructive agricultural insect pests and a major impediment to international fresh commodity trade. The genetic structuring of the species across its entire geographic range has never been undertaken, because under a former taxonomy *B. dorsalis* was divided into four distinct taxonomic entities, each with their own, largely non‐overlapping, distributions. Based on the extensive sampling of six a priori groups from 63 locations, genetic and geometric morphometric datasets were generated to detect macrogeographic population structure, and to determine prior and current invasion pathways of this species. Weak population structure and high genetic diversity were detected among Asian populations. Invasive populations in Africa and Hawaii are inferred to be the result of separate, single invasions from South Asia, while South Asia is also the likely source of other Asian populations. The current northward invasion of *B. dorsalis* into Central China is the result of multiple, repeated dispersal events, most likely related to fruit trade. Results are discussed in the context of global quarantine, trade, and management of this pest. The recent expansion of the fly into temperate China, with very few associated genetic changes, clearly demonstrates the threat posed by this pest to ecologically similar areas in Europe and North America.

## 1 INTRODUCTION

The horticultural sector is one of the largest within the global agricultural economy. Global fruit and vegetable production was estimated at 676.9 and 879.2 million tonnes, respectively, in 2013, with global fruit exports in the same year valued at USD 97.02 billion (Gyan Research and Analytics, 2014). While developed nations such as the United States have long‐established horticultural sectors, significant growth in commercial horticulture is also occurring in the developing world. An expanded horticulture sector is seen not only as a mechanism to increase human health in developing nations, but also as a way of increasing general living standards through cash generation from the sale of fresh commodities for export (Virchow & Jaenicke, 2016; Weinberger & Lumpkin, 2007).

One of the major biological impediments to horticultural production and export in tropical and subtropical regions of the world is the frugivorous tephritid fruit flies (Diptera: Tephritidae) (Hendrichs, Vera, De Meyer, & Clarke, 2015). The tephritid or “true” fruit flies (not to be confused with drosophilid fruit flies) lay their eggs into sound, near‐ripe fruit on plant, where the resultant larvae feed. Depending on commodity, and in the absence of controls, fruit fly damage can easily lead to 80% to 100% crop loss (White & Elson‐Harris, 1992). The global fruit fly problem is exacerbated by a small group of highly polyphagous, highly invasive pest species which competitively dominate local fauna if they enter and establish in a region (Duyck, David, & Quilici, 2004; Duyck et al., 2006), and which can subsequently stop fresh commodity trade because of the quarantine risk they pose (Dohino et al., 2016). The two best known of these invasive tephritids are the Mediterranean fruit fly, *Ceratitis capitata* (Wiedemann), and the focus of this paper, the Oriental fruit fly, *Bactrocera dorsalis* (Hendel).

Oriental fruit fly is one of the world's most invasive and polyphagous pests of agriculture, with a recorded host range of over 250 fruits and vegetables (Clarke et al., 2005). Endemic to the Indo‐Asian region, the fly first established outside this native range in Hawaii in 1945, where it remains a major pest (Vargas, Piñero, & Leblanc, 2015). The fly has subsequently invaded the continental United States on numerous occasions and, while the formal regulatory position is that it is currently absent from the continental United States, debate exists in the scientific literature as to whether it is permanently established in California (Papadopoulos, Plant, & Carey, 2013), or is a repeat invader (Barr et al., 2014). Regardless of the position in the United States, *B. dorsalis* is invasive and permanently established in several South Pacific countries (Vargas et al., 2015), has invaded and been eradicated twice in Australia (Cantrell, Chadwick, & Cahill, 2002), is currently actively invading Central China (Chen, Zhang, Ji, Yang, & Zheng, 2014), and is an “A1” quarantine pest for the European Union (EPPO, 2015). However, it is its invasion, spread, and establishment in sub‐Saharan Africa that has received most attention in recent time. The fly was first detected in Kenya in 2003 (Lux, Copeland, White, Manrakhan, & Billah, 2003), and within a span of 14 years has spread across all of sub‐Saharan Africa and only small parts of South Africa remain free of the pest (Manrakhan, Venter, & Hattingh, 2015). The cost of lost export markets to Africa due to the invasion has been estimated at $2 billion (Ekesi, De Meyer, Mohamed, Massimiliano, & Borgemeister, 2016).

All facets of research and management of this pest have been confounded by its confused taxonomic history, with the fly in recent decades being known under the name of not only *B. dorsalis*, but also *B. invadens* Drew, Tsuruta & White, *B. papayae* Drew & Hancock and *B. philippinensis* Drew & Hancock. As a result of a major international collaborative effort (De Meyer et al., 2015), these latter three species are now recognized as junior synonyms of *B. dorsalis* (Drew & Romig, 2013; Schutze, Mahmood et al., 2015; Schutze, Aketarawong et al., 2015). While the synonymization clarifies taxonomic identity and helps some aspects of pre‐ and post‐harvest control and market access (Dohino et al., 2016; Hendrichs et al., 2015), they also create new challenges. An organism, whose geographic range extends from Africa, across Asia to the Pacific, might be predicted to exhibit macrogeographic population structuring (Ascunce et al., 2011; Gloria‐Soria et al., 2016; Virgilio, Delatte, Backeljau, & De Meyer, 2010; Zhang, Edwards, Kang, & Fuller, 2014). As the International Plant Protection Convention (FAO, 2011) recognizes “Pest” as “any species, strain or biotype of plant, animal or pathogenic agent injurious to plants or plant products,” synonymization of taxa does not negate the issue that different geographic populations may still show high levels of population structuring and so be of potential quarantine and trade concern at the “strain” level.

To address this issue, this paper presents the most comprehensive global assessment of *B. dorsalis* population structuring yet undertaken. We sampled from across the entire range of *B. dorsalis* occurrence, including invasive locations, and used morphological data (geometric morphometric analysis of wing shape) and molecular markers (*cox1* and *nad6* genes and microsatellite loci) to determine global population structuring in this species. Three independent markers were used in an integrative framework (Schlick‐Steiner et al., 2010), and in alignment with previous studies of *B. dorsalis* that have shown these markers to be informative at different temporal and spatial scales of population structuring and invasion biology (Boontop, Schutze, Anthony, Cameron, & Krosch, 2017; Schutze, Krosch et al., 2012; Shi, Kerdelhue, & Ye, 2012). Using DIYABC analysis, our global population data also allow us to make informed comment on the likely origin of *B. dorsalis* within the Indo/Asian region (an issue under debate (Choudhary, Naaz, Prabhakar, & Lemtur, 2016)) and global invasion pathways.

A second major component of our study is to document morphological and genetic changes associated with the current, ongoing northward invasion of *B. dorsalis* into Central China. Although well‐documented in tropical and sub‐tropical China (Wan, Nardi, Zhang, & Liu, 2011), *B. dorsalis* was historically absent from Central China because of climatic unsuitability (specifically cold stress (Stephens, Kriticos, & Leriche, 2007; De Villiers et al., 2016)). Nevertheless, *B. dorsalis* is now able to successfully overwinter in central Chinese provinces, such as Hubei Province (Han et al., 2011). This poses a great concern not only for China, but must also to temperate Europe and North America. Climate models predict these regions to be “unsuitable” for *B. dorsalis* (De Villiers et al., 2016) but, given the Chinese situation, must now be considered at threat. Understanding how this invasion is progressing, whether there is ongoing gene flow with the source population/s, and whether there are morphological or molecular characteristics associated with the invasion front, can help inform management and prevention of novel invasions into Europe and North America. With intensive sampling in China, we assess whether there are morphological and/or genetic signatures associated with the invasion front in China which might help inform the risk posed by this invasive population.

Taken together, we address the following specific research questions with these comprehensive data: (a) whether macrogeographic population structure is detectable in *B. dorsalis* populations across its entire range; (b) what is the likely region of origin for the species and its associated global invasive pathways; and (c) what variation occurs in invasive Central China populations. The results are discussed with respect to the management of this pest.

## 2 MATERIALS AND METHODS

### 2.1 Sample collection

**Figure 1:**
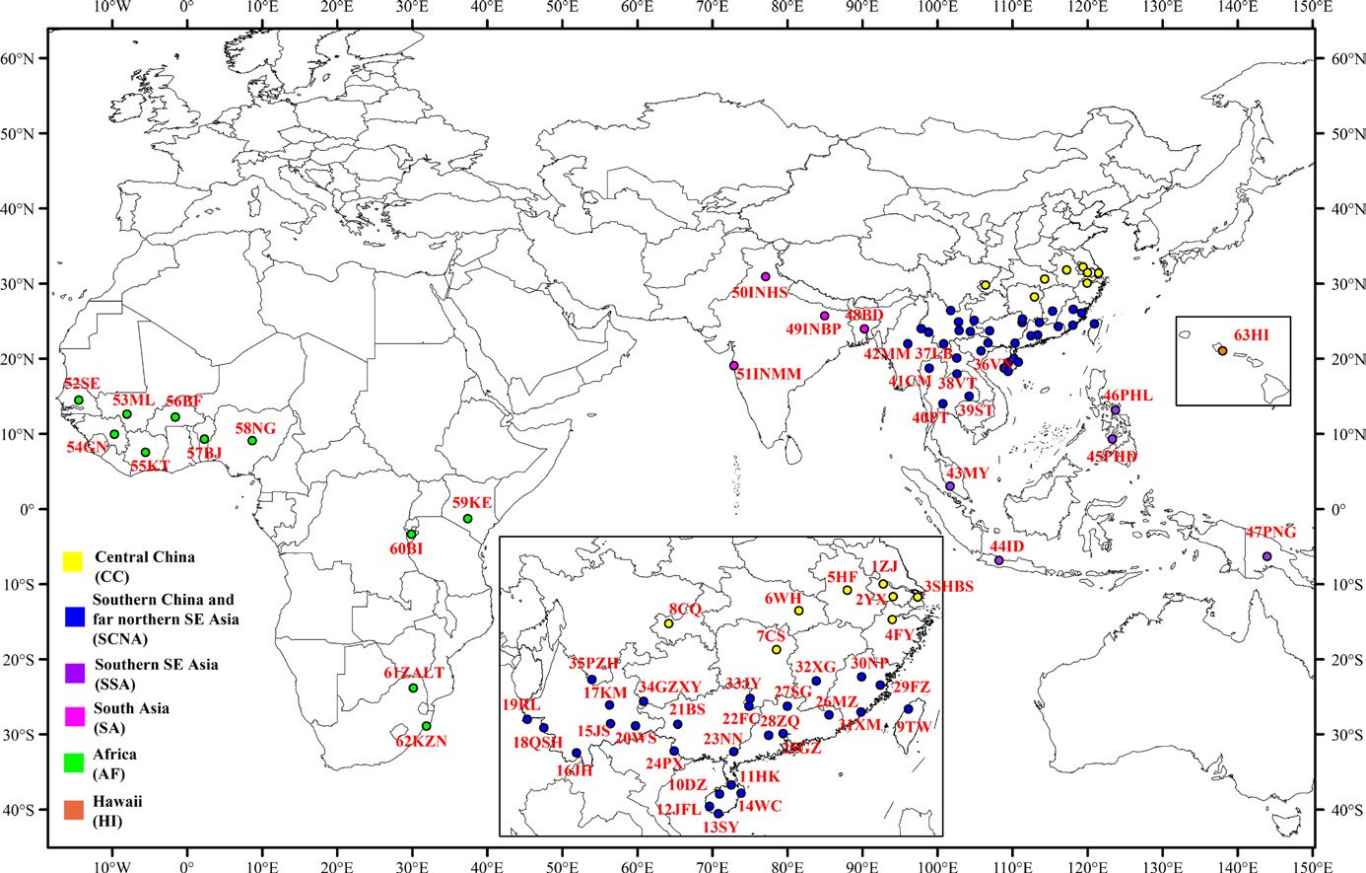
Map showing the sampling sites of 63 populations of *Bactrocera dorsalis*. Specific collection data are presented in Supporting Information Table S1. Note: Insert figure: China, Hawaii. The map was created in ArcGIS 10.2 software (ESRI Inc., Redlands, CA, USA). URL http://www.esri.com/sofware/arcgis/arcgis-for-desktop

Two‐thousand eight‐hundred and sixty‐seven *B. dorsalis* adults were collected from 63 locations and assigned into six a priori groups (Central China (CC), Southern China and far northern South‐East Asia (SCNA), Southern South‐East Asia (SSA), South Asia (SA), Africa (AF) and Hawaii (HI)) based on biogeographical factors. Thirty‐five locations were sampled within China, belonging to 16 provinces. Details of the sampling sites and sampling sizes are given in Supporting Information Table S1, and the localities are shown in Figure 1. All samples were identified using available taxonomic keys prior to conducting molecular analyses (Liang, Yang, Liang, Situ, & Liang, 1996; White & Elson‐Harris, 1992). The sampling involved the use of male‐only lures, so females were not collected. The legs or a portion of the body were removed for genetic analysis and one wing (usually the right) for geometric morphometric shape analysis. The rest of the body and the DNA were stored at −20°C with voucher references for morphological and molecular verification at the Plant Quarantine and Invasion Biology Lab in China Agricultural University.

### 2.2 Geometric morphometric analysis

Usually, the right wing was dissected from each fly for slide mounting, image capture and analysis, the left was used instead if the right wing was damaged. Wings were slide mounted using DPX mounting agent and air‐dried prior to image capture using an AnMo Dino‐Eye microscope eyepiece camera (model # AM423B) mounted on a Leica MZ6 stereomicroscope. Fifteen wing landmarks were selected following Schutze, Jessup, & Clarke, 2012 and digitization using tpsDIG2 v2.16 (Rohlf, 2010).

Raw landmark coordinate data were imported into the computer program MORPHOJ v1.04a (Klingenberg, 2011) for shape analysis. Data were first subjected to Procrustes superimposition to remove all but shape variation (Rohlf, 1999). Multivariate regression of the dependent wing shape variable against centroid size (independent variable) was conducted to assess the effect of wing size on wing shape (i.e., allometry) (Drake & Klingenberg, 2008; Schutze, Jessup et al., 2012). The statistical significance of this regression was tested by permutation tests (10,000 replicates) against the null hypothesis of independence. Subsequent analyses used the residual components as determined from the regression of shape on centroid size to correct for allometric effect.

The size of each wing (centroid size) was calculated in MORPHOJ v1.04a. Centroid size is an isometric estimator of size calculated as the square root of the summed distances of each landmark from the center of the landmark configuration. ANOVA with post hoc Tukey's test was used to assess for significant differences among sample sites.

Samples were a priori assigned to the above six groups (as for centroid size analysis) and 16 provinces within China, from which subsequent canonical variates analysis (CVA) was applied to determine relative differences in wing shape among groups (Krosch et al., 2013). Significant differences were determined via permutation tests (1000 permutation rounds) for Mahalanobis distances among groups. We regressed geographic distance (km) between each pair of sampling sites determined by Google Earth 7.1.7.2606 against Mahalanobis distances calculated from CVA to test for “isolation‐by‐distance” (IbD) effects (Wright, 1943). The strength of the association was determined by linear regression analysis using the program SPSS v17.0.

### 2.3 Genetic analysis

Total genomic DNA was extracted from each individual fly following the manufacturer's protocol from the TIANamp Genomic DNA kit (DP304, TIANGEN, China) for animal tissue, and slight modifications were made to increase DNA concentration (Jiang et al., 2013). Eleven microsatellite loci were used in this study: MS3, MS3B, MS4, MS5, MS6, Bd9, Bd19, Bd42, Bp198, Bp200, Bi5; technical details are given in Dai, Lin, and Chang (2004), Aketarawong, Bonizzoni, Malacrida, Gasperi, and Thanaphum (2006), Shearman et al. (2006) and Khamis et al. (2008). Fluorescently labeled fragments were visualized on ABI PRISM 377 Genetic Analyzer with ROX‐500 size standard. Allele size was analyzed by software GeneScan version 3.7 (Applied Biosystems, Beijing, China).

Gene amplification and sequencing methods were reported previously (Jiang et al., 2013). The primers designed for this study are shown in Supporting Information Table S2. Both directions of the *cox1* (divided into two fragments at first) and *nad6* sequences from each individual were reviewed using Chromas (version 2.33) and assembled using DNAMAN 5.2 (Lynnon Corporation, Quebec, Canada). To delete low‐quality sections, all sequences were aligned with the standard sequences of *B. dorsalis* from NCBI using MEGA 7.0 (Kumar, Stecher, & Tamura, 2016) to generate 1,488‐bp *cox1* sequences and 525‐bp *nad6* sequences, 4,868 sequences were deposited in GenBank with accession numbers MG687532‐MG689973 for *cox1* and MG689974‐MG692399 for *nad6*.

#### 2.3.1 Marker characteristics and intra‐population genetic diversity

For microsatellite data, the number of alleles (*N*_A_), number of effective alleles (*N*_E_), observed heterozygosity (*H*_O_), and expected heterozygosity (*H*_E_) were calculated using POPGENE 1.32 (Yeh, Yang, & Boyle, 1999). Allelic richness (AR) and gene diversity (HS) were calculated using FSTAT 2.9.3.2 (Goudet, 2002). Frequency of null allele (AN) was estimated using GENEPOP 4.1 (Raymond & Rousset, 1995).

For the sequences, the nucleotide composition and variable positions were visualized using MEGA 7 (Kumar et al., 2016). The nucleotide diversity (*π*), haplotype diversity (*Hd*), and the number of haplotypes were estimated using DnaSP 6 (Rozas et al., 2017).

#### 2.3.2 Population genetic structure

Pairwise *F*_ST_ was calculated for both types of markers using Arlequin 3.5 (Excoffier & Lischer, 2010) to measure the degree of genetic differentiation between pairs of populations. We grouped populations into the six prior groups to explore differences between specific regions. Isolation by distance (IBD) was examined by testing the correlation between *F*_ST_ and geographic distances using the program SPSS v17.0.

Bayesian clustering of individuals based on microsatellite genotypes was performed in STRUCTURE 2.0 (Pritchard, Stephens, & Donnelly, 2000) to infer genetic structure among the six a priori defined groups and 63 populations of *B. dorsalis*. We set the number of clusters (*K*) from 1 to 10 and conducted 10 independent runs for each value of *K*. Each run consisted of a burn‐in period of 50,000 steps, followed by 100,000 Markov chain Monte Carlo (MCMC) repetitions with a model allowing admixture. Δ*K* values (Evanno, Regnaut, & Goudet, 2005) were computed to select the most likely number of *K* using the online resource Structure Harvester (Earl, 2011) that explained the structure in data. We then conducted model to summarize cluster membership coefficient matrices for each value of *K* with CLUMPP 1.1.2 (Jakobsson & Rosenberg, 2007), and plotted using DISTRUCT 1.1 (Rosenberg, 2004).

Evolutionary relationships among *cox1* and *nad6* haplotypes were inferred using a haplotype network, constructed under the median‐joining (MJ) method in NETWORK 5.0.0.3 (Bandelt, Forster, & Rohl, 1999).

#### 2.3.3 Demographic history

DIYABC 2.1 (Cornuet et al., 2014) was used to test evolutionary scenarios of expansion: nine scenarios were examined for 2867 individuals divided into South Asia (SA), Rest Asia (CC, SCNA and SSA), Africa, and Hawaii to test invasive pathways and hypotheses of whether the likely region of origin is South Asia or Rest Asia. A generalized stepwise model was used, with a gamma distribution on the mutation rate and default values for all other parameters (Boontop et al., 2017). Ninety thousand simulated datasets were computed, and posterior probabilities for each scenario were assessed using both the direct and logistic regression methods and the closest 90,000 simulated datasets to the observed data. Rates of type I and II error were estimated as a measure of confidence in scenario choice.

Neutrality tests and mismatch distribution of the sequences were calculated in Arlequin 3.5 with 1,000 bootstrap replicates, six parameters were calculated: effective population size before expansion (*Ɵ*_0_), effective population size after expansion (*Ɵ*_1_), time of populations expansion (*T*), Tajima's *D*, Fu's *Fs* and sum of square deviation (SSD) between expected and observed mismatch distribution.

## 3 RESULTS

### 3.1 Geometric morphometric analysis

**Figure 2:**
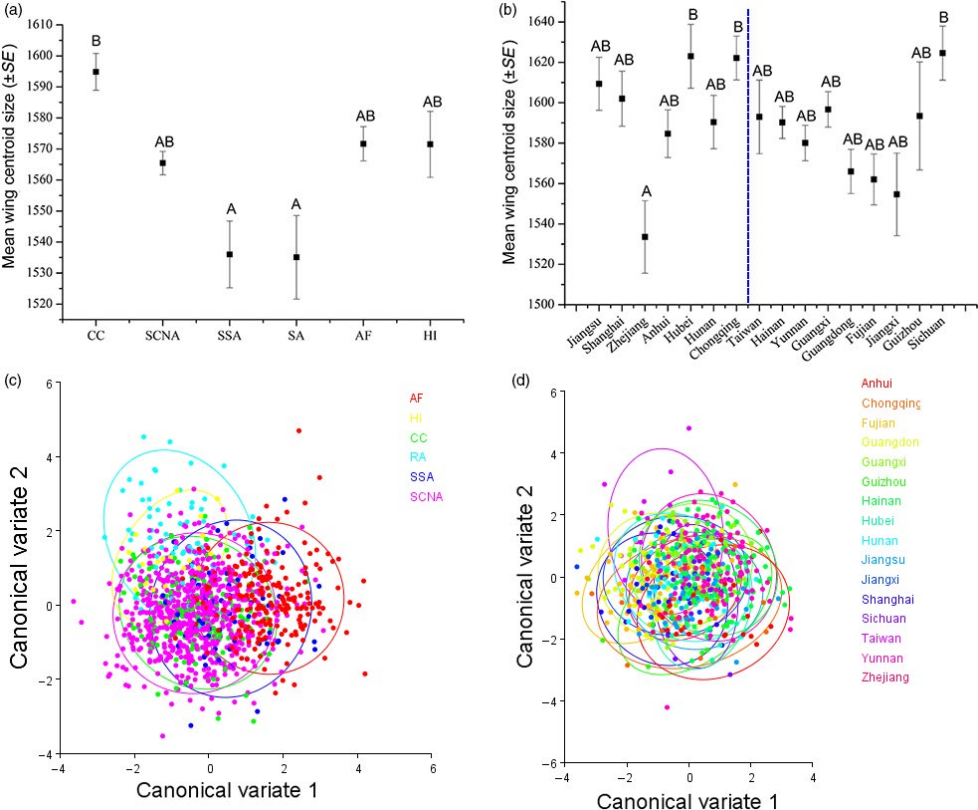
Morphometric results for centroid size and wing shape analysis of *Bactrocera dorsalis*. (a) Mean (±SE) wing centroid size from six groups and (b) 16 provinces/cities, the blue dotted line divides central and southern China. Samples sharing the same letter are not statistically different from each other based on one‐way ANOVA with Turkey's post hoc test (*α* = 0.05). (c) plot of the first two variates following canonical variate analysis from six groups and (d) 16 provinces/cities

One‐thousand two‐hundred and sixteen wings from flies collected from 63 locations covering six a priori defined geographic groupings (Figure 1 and Supporting Information Table S1) were used for geometric morphometric analysis. Wing centroid size varied significantly across the six population groups (*F*_5, 1210_ = 6.128; *p* < 0.05), with significant variation found between populations from Central China (largest wings) compared with South Asia and southern South‐East Asia (smallest wings): The three remaining population groups were intermediate between, and not significantly different from, the two extremes (Figure 2a). Within China, flies from Hubei and Chongqing (Central China), and Sichuan (southern China) possessed significantly larger wings than those from Zhejiang province (Central China), but all other populations were not significantly different from each other (*F*_15,668_ = 2.427; *p* < 0.05) (Figure 2b).

Canonical variate analysis for the six groups and 16 provinces/cities within China produced five and 15 canonical variates respectively, of which the first two canonical variates cumulatively explained 76.29% and 44.49% of the variation. However, there was no evidence for structuring among groups in the CVA plots (Figure 2c,d). In contrast, among‐group Mahalanobis distances were significantly different for all six groups and most Chinese provinces/cities. Hawaii showed the greatest differentiation from other groups, with the greatest Mahalanobis distance found between Hawaii and Africa. Within China, Taiwan showed significant differentiation from all other provinces/cities, with the largest Mahalanobis distance occurring between Taiwan and Anhui (Central China). Invasive Chinese populations (i.e., central Chinese populations) were not noticeably different from southern Chinese populations (Table 1). For the full dataset, a significant isolation‐by‐distance relationship (Pearson correlation = 0.514; *p* < 0.01) was detected between geographic distances and Mahalanobis distances (Supporting Information Figure S1).

**Table 1:** Mahalanobis distances between six groups and 16 provinces/cities

	Central China (CC)	Southern China and far northern SE Asia (SCNA)	Southern SE Asia (SSA)	South Asia (SA)	Africa (AF)										
SCNA	**0.7781**	**–**													
RA	**2.2706**	**2.1703**	**–**												
SA	**1.721**	**1.6021**	**2.6807**	**–**											
AF	**2.0438**	**2.0525**	**2.9915**	**1.7713**	**–**										
HI	**3.0792**	**3.0424**	**3.1369**	**3.6337**	**3.8003**										

	Jiangsu	Shanghai	Zhejiang	Anhui	Hubei	Hunan	Chongqing	Taiwan	Hainan	Yunnan	Guangxi	Guangdong	Fujian	Jiangxi	Guizhou
Jiangsu															
Shanghai	**1.9141**														
Zhejiang	**1.8374**	**1.9686**													
Anhui	**1.6679**	**2.4412**	**2.0452**												
Hubei	**1.6718**	**2.0538**	**2.0677**	1.4068											
Hunan	**1.7115**	**1.6941**	1.5851	**2.124**	**1.8989**										
Chongqing	1.5182	1.5887	1.6929	1.667	1.5831	1.5801									
Taiwan	**2.5583**	**2.8036**	**2.9027**	**3.4275**	**3.116**	**2.4882**	**2.9934**								
Hainan	**1.7207**	**2.2354**	**1.8099**	**1.9817**	1.5556	**1.4957**	**1.8494**	**2.5907**							
Yunnan	**1.5236**	**2.2307**	**1.8728**	**1.849**	**1.9745**	**1.6736**	**1.6663**	**2.557**	**1.5081**						
Guangxi	**1.6151**	**2.1734**	1.5159	**2.0035**	**1.9151**	1.1692	**1.6712**	**2.4013**	**1.3197**	**1.1678**					
Guangdong	**1.7677**	**1.7435**	**1.6391**	**2.3417**	**2.0809**	**1.4378**	**1.7874**	**2.4531**	**1.9758**	**1.8538**	**1.2385**				
Fujian	**2.0611**	1.5594	**2.0949**	**2.6206**	**2.3174**	**1.8426**	**1.965**	**2.4305**	**2.2028**	**2.2209**	**1.8848**	**1.2985**			
Jiangxi	**1.9037**	1.9589	**1.9932**	**2.2396**	**2.1276**	1.3178	1.7335	**2.305**	**1.9974**	**2.0147**	1.5828	**1.75**	**2.0477**		
Guizhou	1.6268	**2.0175**	**1.8744**	1.7615	1.7833	1.6076	1.3603	**3.0676**	**2.0522**	**1.7394**	**1.7717**	**1.9452**	**2.2893**	**2.098**	
Sichuan	**1.7946**	**2.3149**	**1.8899**	1.7168	**1.9387**	**1.9976**	**2.1154**	**2.8587**	**2.0013**	**1.9728**	**1.8997**	**2.1301**	**2.3108**	**2.2094**	1.7486

Note

Values in bold are significant at *p* < 0.005; the dotted line divides central and southern China.

### 3.2 Genetic analysis

#### 3.2.1 Marker characteristics and intra‐population genetic diversity

From a total of 2,867 *B. dorsalis* individuals screened for 11 microsatellite loci, 236 alleles were observed ranging from 15 to 36 per locus. Higher genetic diversity (*H*_S_) was found in all Asian groups (CC, SCNA, SSA, SA–average *H*_S_ = 0.632), whereas invasive populations in Africa (*H*_S_ = 0.547) and Hawaii (*H*_S_ = 0.413) had lower genetic diversity (Table 2, Supporting Information Table S3). In addition to nuclear microsatellites, the mitochondrial genes, *cox1* (1,488 bp) and *nad6* (525 bp) were sequenced from 2,442 (1,284 haplotypes) and 2,426 (609 haplotypes) individuals, respectively. Both mitochondrial genes exhibited greater haplotype and nucleotide diversity in the Asian groups than Africa and Hawaii (Table 2, Supporting Information Table S3). All 35 Chinese populations showed high genetic diversity for all molecular markers (Supporting Information Table S3).

**Table 2:** Genetic diversity indices in six groups of *Bactrocera dorsalis*

	Code	SSR								*cox1*					*nd6*				
		Size	*N*_A_	*N*_E_	*H*_O_	*H*_E_	*A*_R_	*A*_N_	*H*_S_	Size	N	*Hd*	*π*	*k*	Size	N	*Hd*	*π*	*k*
1	CC	379	12.636	3.223	0.463	0.634	8.217	0.107	0.635	318	174	0.981	0.00630	9.375	325	121	0.956	0.00797	4.183
2	SCNA	1528	19.273	3.550	0.481	0.659	9.000	0.110	0.659	1390	905	0.993	0.00725	10.783	1316	445	0.965	0.00798	4.19
3	SSA	219	11.182	2.997	0.442	0.616	7.649	0.113	0.616	203	116	0.975	0.01059	15.746	191	60	0.870	0.01129	5.929
4	SA	178	9.818	3.344	0.507	0.618	7.889	0.070	0.618	156	114	0.994	0.00742	11.043	159	76	0.933	0.00752	3.947
5	AF	513	10.455	2.682	0.425	0.547	5.739	0.093	0.547	337	45	0.673	0.00452	6.727	385	12	0.603	0.00339	1.778
6	HI	50	3.182	2.298	0.313	0.412	3.182	0.083	0.413	38	8	0.636	0.00148	2.209	50	3	0.496	0.00187	0.98

Note

*A*_N_: mean frequency of null alleles; *A*_R_: mean allelic richness; *Hd*: haplotype diversity; *H*_E_: mean expected heterozygosity; *H*_O_: mean observed heterozygosity; *H*_S_: gene diversity; *k*: average numbers of nucleotide differences; N: number of haplotypes in each population; *N*_A_: mean number of alleles; *N*_E_: mean number of effective alleles; *π*: nucleotide diversity.

#### 3.2.2 Population genetic structure

Significant genetic differentiation based on among‐site *F*_ST_ indices was observed between all regions and across all molecular markers (Table 3, Supporting Information Tables S4 and S5). Hawaii showed the greatest *F*_ST_ differentiation with all other sites, whereas all other comparisons among the other regions demonstrated much lower, albeit still significant, variation (Table 3). Isolation by distance (IBD) was detected from the two sets of molecular markers (Supporting Information Figure S1). Within China, genetic differentiation was low among all sample sites (Supporting Information Tables S4 and S5).

**Table 3:** Pairwise *F*_ST_ among six groups of *Bactrocera dorsalis*

	CC	SCNA	SSA	SA	AF	HI
SSR						
SCNA	**0.00539**	**–**				
SSA	**0.03269**	**0.02986**	**–**			
SA	**0.01698**	**0.01245**	**0.02651**	**–**		
AF	**0.05488**	**0.05217**	**0.0772**	**0.04672**	**–**	
HI	**0.12552**	**0.12985**	**0.14766**	**0.11694**	**0.13068**	**–**
*cox1/nd6*						
CC	**–**	**0.00261**	**0.14469**	**0.01636**	**0.16363**	**0.53646**
SCNA	**0.00426**	**–**	**0.15011**	**0.01387**	**0.13823**	**0.51927**
SSA	**0.12419**	**0.10505**	**–**	**0.14968**	**0.32940**	**0.50660**
SA	**0.01916**	**0.00813**	**0.08465**	**–**	**0.13246**	**0.56882**
AF	**0.05488**	**0.21112**	**0.25283**	**0.19134**	**–**	**0.69480**
HI	**0.57409**	**0.50943**	**0.41203**	**0.53441**	**0.69314**	

Note

Values in bold are significant at *p *<* *0.05.

**Figure 3:**
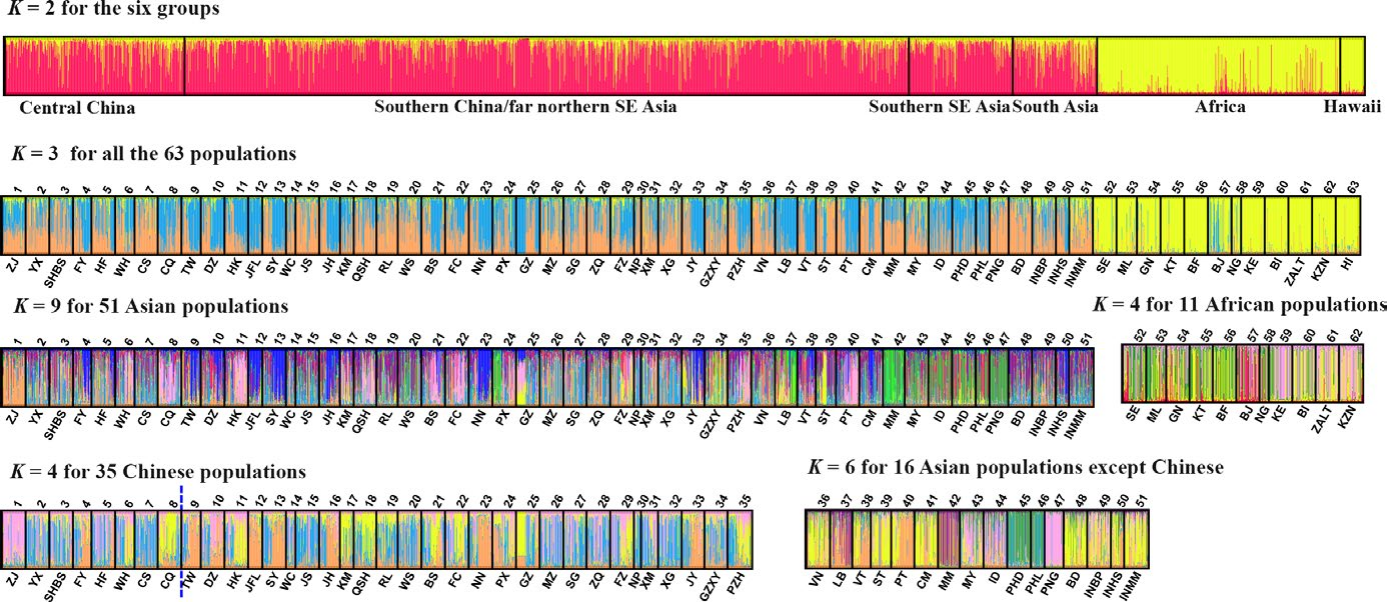
Bayesian results based on STRUCTURE of *Bactrocera dorsalis*. Individuals were grouped by six groups or 63 collection site according to Figure 1 and Supporting Information Table S1, each individual was represented by a vertical bar displaying membership coefficients, and the blue dotted line divides central and southern China

Bayesian clustering analysis of microsatellite genotypes implemented in STRUCTURE suggested that the maximum value for the estimated likelihood of *K* was found at *K* = 2 for the six group dataset and *K* = 3 for all 63 populations. Visualization of cluster assignments indicated African locations, and Hawaii formed a cluster separate to all other Asian locations. No structure was observed within Asia when analyzed with African and Hawaiian populations (Figure 3). Separate analyses of Asian, Chinese, and African populations were conducted to explore population structure within these regions (Figure 3). *K* = 4 was found for African populations where the western African populations were grouped except for Benin (BJ) which formed a unique cluster; Burundi (BI), South Africa (ZALT, KZN), and most of Kenya (KE) formed another cluster. *K* = 9 showed a complex structure for 51 Asian populations; *K* = 4 and *K* = 6 were found for the 35 Chinese populations and the other 16 Asian populations, respectively. Zhenjiang (ZJ) from Central China alone was separated from the other three clusters within China; while Myanmar (MM), the Philippines (PHD, PHL), and Papua New Guinea (PNG) were differentiated from the other Asian populations.

**Figure 4:**
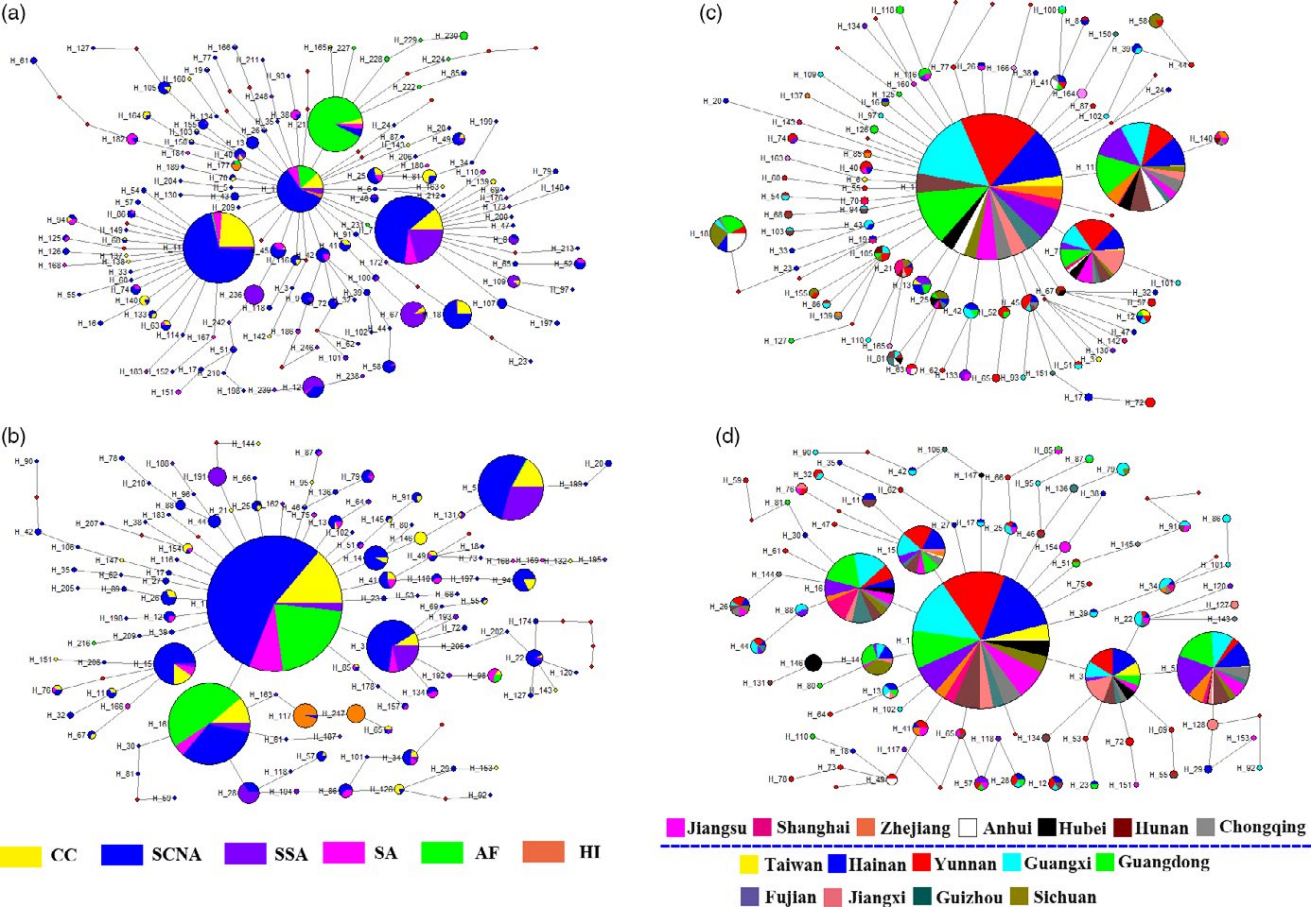
Median‐Joining haplotype network of *Bactrocera dorsalis* for six groups and 16 provinces/cities based on *cox1* (a,c) and *nad6* (b,d) data. Size of nodes and pie segments were proportional to haplotype frequency, H1 (contained 1,141 sequences) from Figure 3A only displayed the proportion of the six groups by the software, the small black circles represent median vectors (roughly equivalent to hypothetical unsampled haplotypes), length of the branched is proportional to number of mutational changes between haplotypes, and the blue dotted line divides central and southern China

Relationships among mitochondrial haplotypes were inferred using network analysis, which showed largely starlike patterns for both *cox1* (249 haplotypes) and *nad6* (219 haplotypes) without the third base of codons, and only the torso was displayed (Figure 4). All six groups possessed several high‐frequency shared haplotypes, including many that were central to the network (Figure 4a,b). Further, the four Asian groups showed high haplotype diversity relative to Africa and Hawaii. There was no evidence for macrogeographic population structure among regions. Similarly, there was no apparent structure observed within China, with many haplotypes shared among provinces/cities, and with all locations showing similarly high haplotype diversity (Figure 4c, d).

#### 3.2.3 Demographic history

Nine scenarios were assessed in DIYABC analysis (Supporting Information Figure S2) to identify the likely origin of *B. dorsalis* within the Indo/Asian region and infer global invasion pathways. Logistic regression highly supported a scenario of stepwise expansion of *B. dorsalis* from South Asia to Rest Asia and subsequently Hawaii, then Africa (scenario 3, *p* = 0.93, Supporting Information Figures S2 and S3), with confidence intervals that did not overlap with other scenarios (Table 4). Global posterior predictive error under the logistic approach was 0.474, suggesting the correct scenario was supported in 526 of 1,000 test datasets, whereas the global prior predictive error was 0.516. The type I error for the selected scenario (the proportion of 1,000 test datasets in which this scenario was incorrectly rejected) was 0.571. Taken together, this supports the notion that South Asia is the center of origin for *B. dorsalis*.

**Table 4:** The confidence intervals of direct estimate and logistic regression for chosen scenarios

Scenario	Direct method posterior probability (*nδ* = 500)	95% Confidence Intervals	Logistic regression posterior probability (*nδ* = 90,000)	95% Confidence Intervals
1	0.0820	0.0000–0.3225	0.0375	0.0000–0.1979
2	0.1100	0.0000–0.3843	0.0010	0.0000–0.1662
3	0.1560	0.0000–0.4741	0.9300	0.9179–0.9421
4	0.0760	0.0000–0.3083	0.0001	0.0000–0.1653
5	0.1080	0.0000–0.3801	0.0011	0.0000–0.1663
6	0.1200	0.0000–0.4048	0.0267	0.0000–0.1881
7	0.0960	0.0000–0.3542	0.0025	0.0000–0.1675
8	0.0640	0.0000–0.2785	0.0001	0.0000–0.1654
9	0.1880	0.0000–0.5305	0.0010	0.0000–0.1750

Neutrality tests performed on the *cox1* and *nad6* dataset produced significant negative Tajima's *D* and Fu's *F*_S_ values for Asian populations. Ratios between estimated effective population size after expansion (*Ɵ*_1_) and effective population size before expansion (*Ɵ*_**0**_), which can serve as an estimate of the extent of population growth, indicated that *B. dorsalis* exhibited a certain degree of population growth in all the groups (Supporting Information Table S6). Furthermore, the unimodal mismatch distribution (Supporting Information Figure S4) supported a model of population expansion (*p*_SSD_ > 0.05).

## 4 DISCUSSION

This study represents the largest‐ever sampling of *B. dorsalis* populations and encompasses both the accepted native range and recent (<70 years) invasive locations. This extensive sampling provides higher resolution of population relationships and more rigorous tests regarding invasive history and region of origin of this highly invasive and pestiferous fruit fly than previously available.

### 4.1 Global population structure

Microsatellite markers divided *B. dorsalis* into two genetic units, an Asian group and a non‐Asian group, while the network of mitochondrial haplotypes did not suggest any subdivision in the data. The non‐Asian group inferred in the microsatellite data corresponded to known recent invasive populations in Africa and Hawaii. Interpretation of this pattern would normally imply recent common ancestry between these locations; however, it is much more likely in this case that the pattern shows the effect of separate founder events in each location (Nardi, Carapelli, Dallai, Roderick, & Frati, 2005; Sakai et al., 2001). Both Africa and Hawaii have much lower molecular diversity than Asia overall, and the two locations are supported by ABC analysis as arising from a South Asian source in separate colonization events. Further, genetic differentiation indices support these populations as significantly different to most Asian locations, and also to each other. Overall, we argue that these data strongly suggest that Hawaii and Africa are the result of single separate invasions, with little or no subsequent gene flow with source populations in South Asia. This supports previous analyses of African populations of *B. dorsalis* (Khamis et al., 2009; Schutze et al., 2015), but suggests also that founder effects in separate invasive populations that arose from the same source can cause these populations to appear similar under some analytical scenarios.

Within Asia, weak genetic structure and/or isolation‐by‐distance trends have been recorded in all previous studies (Aketarawong et al., 2007; Schutze, Krosch et al., 2012; Shi et al., 2012; Wan, Liu, & Zhang, 2012) and were explained by repeated long‐distance migration events, facilitated by the polyphagy of the fruit fly. Our genetic and morphological results within Asia agree with this hypothesis and infer that there is no macrogeographic sub‐structuring across the Asian region. Microsatellite data suggested some individual populations may be slightly differentiated from their neighbors, especially Zhenjiang (ZJ), Myanmar (MM), and Papua New Guinea (PNG). These populations are also characterized by lower overall genetic diversity. The presence of *B. dorsalis* in ZJ and PNG is the result of very recent colonization events at the northeastern and southeastern invasion fronts of *B. dorsalis*, respectively. In contrast, Myanmar would normally be considered part of the native range of *B. dorsalis* in South‐East Asia and populations should express high genetic diversity. The lower diversity there may be owing to trade practices and geographic barriers (Aketarawong et al., 2007; Shi et al., 2012), but this is purely conjectural as there are no obvious explanations for the low genetic diversity recorded for this population.

High levels of genetic diversity and gene flow observed between Asian populations of *B. dorsalis* have historically clouded inferences of its potential region of origin. Northern South‐East Asia or Southern China had been inferred as the potential origin by previous authors (Aketarawong et al., 2007; Li, Wu, Chen, Wu, & Li, 2012; Schutze, Krosch et al., 2012; Shi et al., 2012; Wan et al., 2012); however, these studies lacked critical Indian/South Asian populations. Recent work within India has shown high genetic diversity within Indian populations (*cox 1 Hd* 0.833–1.00), but there was very limited integration of that data with non‐Indian populations (Choudhary et al., 2016). Our global population data allow us to test multiple global invasive pathway scenarios under an ABC framework, and these tests continue to support the hypothesis of South Asia (= India + Bangladesh) as the original location, even with extensive sampling through the rest of Asia.

### 4.2 North‐Central Chinese invasion

Within China, the ongoing northward spread of *B. dorsalis* allows direct tests of morphological and molecular differentiation at the invasion front. This invasion began in the early 2000s and northward expansion continues (Wang et al., 2009; Yuan, Wang, Song, Rong, & Yin, 2008). Critically, *B. dorsalis* is now moving into central areas of China which are climatically similar to temperate regions in Europe and North America, which were previously thought climatically unsuitable to the fly due to overwintering cold stress (De Villiers et al., 2016; Han et al., 2011). In this, *B. dorsalis* is proving very similar to *B. tryoni* (Froggatt), another tropical fruit fly species which has demonstrated the capacity to survive temperate winters (Meats, 1976; O'Loughlin, East, & Meats, 1984). Understanding how the *B. dorsalis* invasion is progressing, whether there is ongoing gene flow with the source population/s, and whether there are morphological or molecular characteristics associated with the invasion front, can help inform management and prevention of novel invasions into Europe and North America.

From the extensive sampling within China conducted here, our data suggest that there are only subtle signatures associated with the invasion front. Central Chinese flies possessed the largest wings of any sampled group and were significantly different to South and South‐East Asian populations; however, they were not significantly different to southern Chinese populations. Larger body size in northern populations may be an adaptation to cooler temperatures, following a Bergmann cline type model (Blackburn, Gaston, & Loder, 1999), but a lack of correlation between wing centroid size and latitude for Chinese populations (analysis not presented) means this is unlikely. Microsatellite data suggest that although there are four population clusters supported within China, none of these correspond to the invasive central populations; it is likely that the frequent fruit trade from southern to northern China facilitates multiple repeated dispersal events. Global climate change may be creating suitable conditions for the northward spread of this species, just as it has for the southward spread of *B. tryoni* into temperate Australia (Sultana, Baumgartner, Dominiak, Royer, & Beaumont, 2017). This ongoing expansion of *B. dorsalis* into regions that were considered climatically unsuitable poses a threat not only to northern China, but also to ecologically similar areas in Europe and North America should it establish on either continent.

### 4.3 Implications for management

Despite our extensive sampling across *B. dorsalis*'s entire geographic range, no macrogeographic population structuring was observed: a result fully consistent with prior studies which have covered individual components of the range (Choudhary et al., 2016; Khamis et al., 2009; Schutze, Krosch et al., 2012; Shi et al., 2012). The genetic uniqueness of Hawaii and Africa is linked to the recent invasions (60–15 years, respectively, assuming the invasive populations were detected soon after their establishment) of those locations, not because of long‐term population structuring. Given such consistent results from multiple studies, we consider it highly unlikely that different geographic populations of the fly will show marked biological differences, such as differences in host use or thermal tolerances, which might impact on quarantine, trade, or pest management. Indeed, where post‐harvest market access research has been done on different geographic populations of *B. dorsalis*, no significant differences have been detected between populations (Hallman, Myers, Jessup, & Islam, 2011; Myers, Cancio‐Martinez, Hallman, Fontenot, & Vreysen, 2016), a result consistent with the lack of population structuring.

The current movement of *B. dorsalis* into Central China, without any apparent strong selective pressure, must pose a deep concern for other temperate regions of the world, especially Europe and North America. The invasion and spread in the last decade of spotted‐wing drosophila, *Drosophila suzukii* Matsumura, across both Europe and North America, has demonstrated just how susceptible the fruit growing industries in those continents are to polyphagous fruit feeders (Lee et al., 2011; Walsh et al., 2011). Yet, as damaging as spotted‐wing drosophila is, its impact is still largely restricted to berries and soft fruits. In contrast, *B. dorsalis* attacks fruits from well over 20 plant families and is generally regarded as one of global agriculture's most damaging insects (Clarke et al., 2005). Should it permanently establish in North Asia, America or Europe, we anticipate a far more northerly spread than currently predicted by climate matching models (De Villiers et al., 2016).

## CONFLICTS OF INTEREST

The authors declare no conflict of interest.

## DATA ARCHIVING STATEMENT

Wing shape data and microsatellite genotypes data for this study are available at Dryad Digital Repository: https://doi.org/10.5061/dryad.kh0f141; DNA sequences: Genbank accessions MG687532‐MG692399.
