# Supplementary material for: Population structure of a global agricultural invasive pest, Bactrocera dorsalis (Diptera: Tephritidae)
Source: Evol Appl. 2018 Sep 28;11(10):1990–2003. doi: 10.1111/eva.12701 (PMC6231469; doi:10.1111/eva.12701)
Supplement: Supplementary file 1 [file EVA-11-1990-s001.docx]

**Supplemental Information for:**

**Population structure of a global agricultural invasive pest, *Bactrocera dorsalis* (Diptera: Tephritidae)**

Yu-jia Qin^1a^, Matthew N. Krosch^2a^, Mark K. Schutze^2^, Yue Zhang^1^, Xiao-xue Wang^1^, Chandra Shekhar Prabhakar^2, 3^, Agus Susanto^4^, Alvin K.W. Hee^5^, Sunday Ekesi^6^, Kemo BADJI^7^, Mahfuza Khan^8^, Yu-bing Huang^9^, Jia-jiao Wu^10^, Qiao-ling Wang^1^, Ge Yan^1^, Li-huan Zhu^1^, Zi-hua Zhao^1^, Li-jun Liu^1^, Anthony R. Clarke^2^, Zhi-hong Li^1^

^a^These authors contributed equally to this work.

Correspondence: Zhi-hong Li, [lizh@cau.edu.cn](mailto:lizh@cau.edu.cn) and Anthony R. Clarke, [a.clarke@qut.edu.au](mailto:a.clarke@qut.edu.au)

**
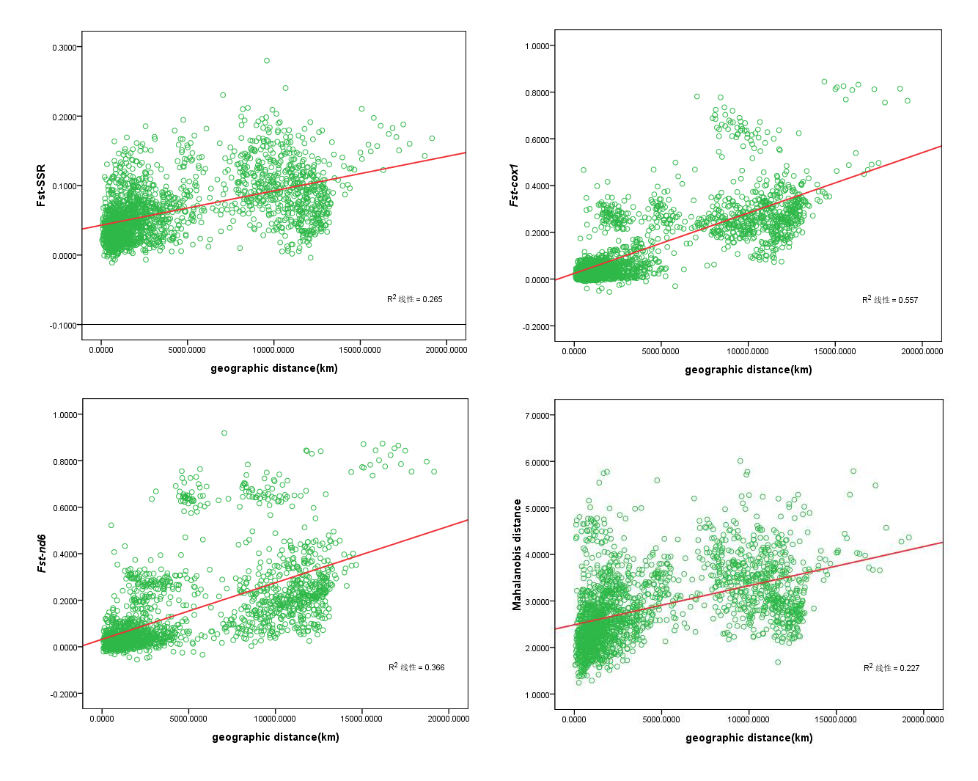
**

Fig. S1 Correlation test based on geometric morphometric and genetic analysis of *B. dorsalis.*


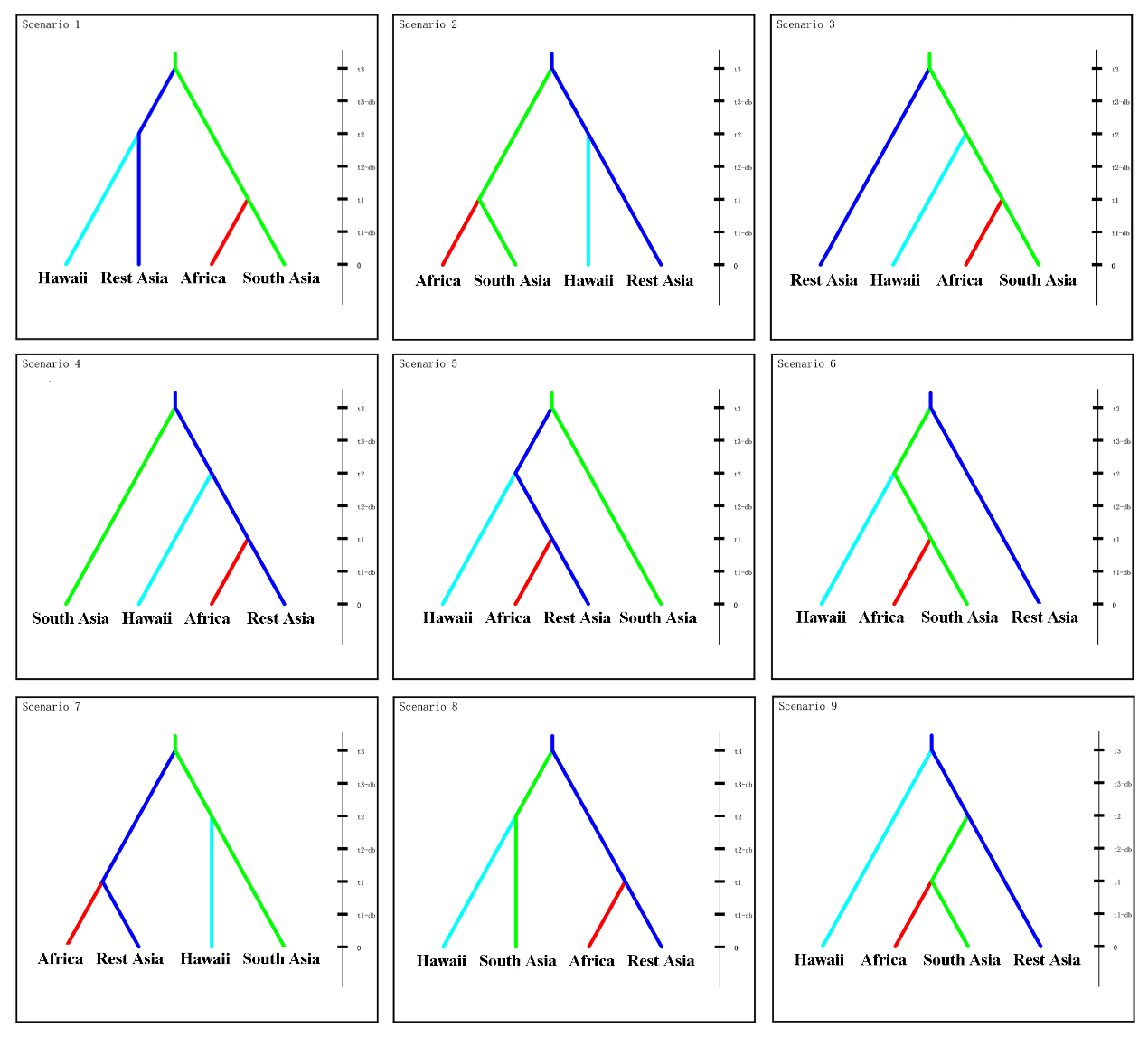


Fig. S2 Evolutionary scenarios of *B. dorsalis* colonization from four groups, evaluated using approximate Bayesian computation inference as implemented by ABC method with time scale (t1, to t3) and bottleneck (db= duration of bottleneck).


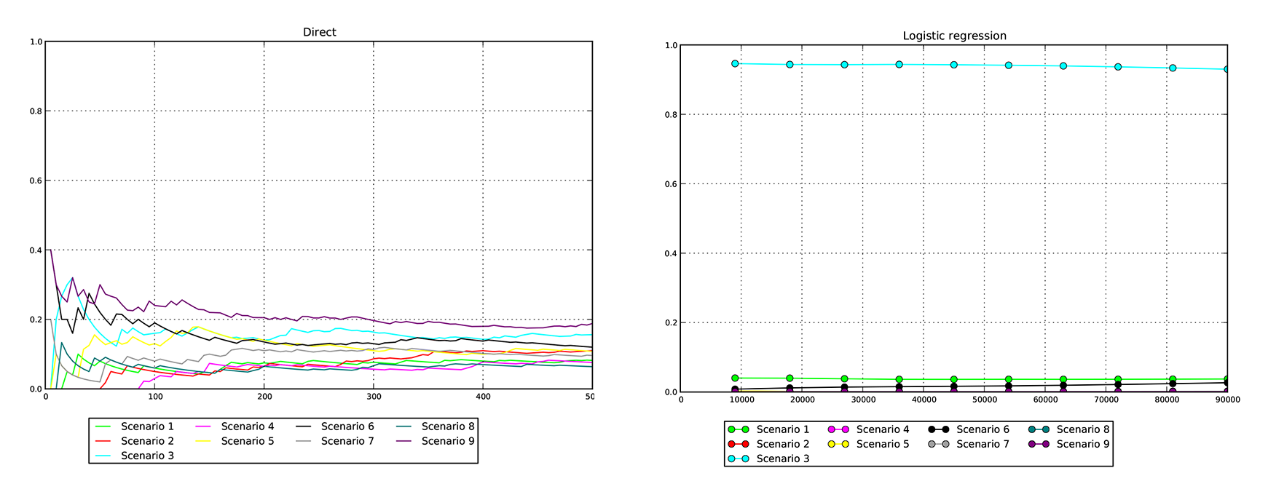


Fig. S3 Plots of scenario posterior probabilities under both the direct and logistic regression methods.


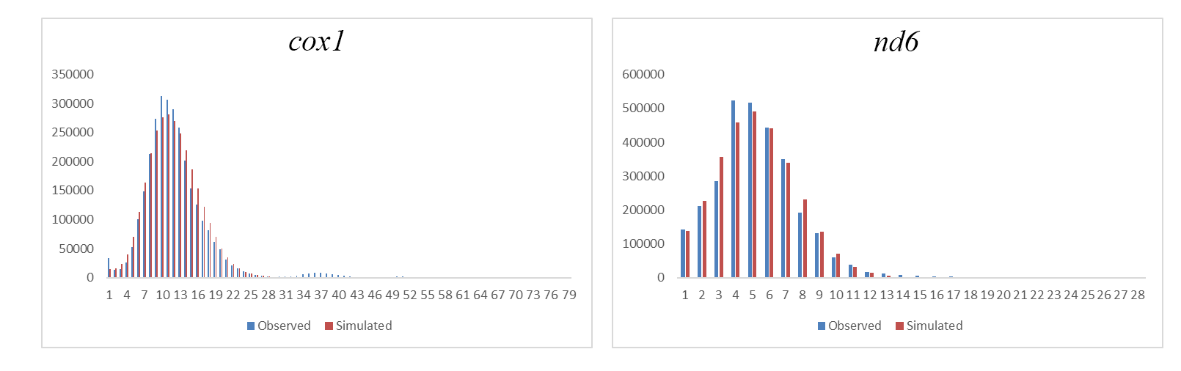


Fig. S4. Mismatch distributions of the sequences of *B. dorsalis.*

Table S1 Sample information of the 63 populations used in this study

| **Area** |  | **Code** | | **Country**  **/Province** | **Collection**  **site** | **Latitude** | **Longitude** | **Year** | | **Sample size** | | | | |
| --- | --- | --- | --- | --- | --- | --- | --- | --- | --- | --- | --- | --- | --- | --- |
|  |  |  |  |  |  |  |  |  | **SSR** | | ***cox1*** | | ***nad6*** | **Wings** |
| **Central**  **China** | 1 | | ZJ | Jiangsu | Zhenjiang | 32.195 | 119.412 | 2014 | | 50 | | 43 | 50 | 20 |
|  | 2 | | YX |  | Yixing | 31.435 | 120.004 | 2012 | | 50 | | 26 | 39 | 20 |
|  | 3 | | SHBS | Shanghai | Baoshan | 31.405 | 121.49 | 2010 | | 50 | | 30 | 47 | 20 |
|  | 4 | | FY | Zhejiang | Fuyang | 30.049 | 119.96 | 2014 | | 39 | | 39 | 39 | 20 |
|  | 5 | | HF | Anhui | Hefei | 31.821 | 117.227 | 2014 | | 50 | | 49 | 10 | 20 |
|  | 6 | | WH | Hubei | Wuhan | 30.593 | 114.305 | 2014 | | 41 | | 41 | 43 | 20 |
|  | 7 | | CS | Hunan | Changsha | 28.228 | 112.939 | 2014 | | 49 | | 42 | 48 | 18 |
|  | 8 | | CQ | Chongqing | Chongqing | 29.806 | 106.414 | 2014 | | 50 | | 48 | 49 | 20 |
| **Southern China/far northern**  **SE Asia** | 9 | | TW | Taiwan | Sanwan | 24.631 | 120.93 | 2014 | | 41 | | 34 | 39 | 21 |
|  | 10 | | DZ | Hainan | Danzhou | 19.5 | 109.483 | 2010 | | 50 | | 20 | 19 | 20 |
|  | 11 | | HK |  | Haikou | 20.044 | 110.198 | 2013 | | 50 | | 48 | 48 | 20 |
|  | 12 | | JFL |  | Jianfengling | 18.75 | 108.883 | 2007 | | 30 | | 48 | 49 | 20 |
|  | 13 | | SY |  | Sanya | 18.311 | 109.414 | 2007 | | 50 | | 30 | 50 | 20 |
|  | 14 | | WC |  | Wencang | 19.543 | 110.798 | 2007 | | 20 | | 20 | 17 | 20 |
|  | 15 | | JS | Yunnan | Jianshui | 23.747 | 102.893 | 2014 | | 50 | | 50 | 28 | 20 |
|  | 16 | | JH |  | Jinghong | 21.983 | 100.833 | 2013 | | 44 | | 43 | 13 | 20 |
|  | 17 | | KM |  | Kunming | 24.88 | 102.833 | 2014 | | 29 | | 31 | 21 | 20 |
|  | 18 | | QSH |  | Qingshuihe | 23.495 | 98.849 | 2013 | | 48 | | 46 | 50 | 19 |
|  | 19 | | RL |  | Ruili | 24.013 | 97.852 | 2013 | | 45 | | 45 | 40 | 18 |
|  | 20 | | WS |  | Wenshan | 23.617 | 104.4 | 2011 | | 50 | | 24 | 29 | 20 |
|  | 21 | | BS | Guangxi | Baise | 23.713 | 106.9595 | 2014 | | 50 | | 47 | 50 | 20 |
|  | 22 | | FC |  | Fuchuan | 24.814 | 111.277 | 2014 | | 50 | | 50 | 47 | 20 |
|  | 23 | | NN |  | Nanning | 22.055 | 110.356 | 2009 | | 50 | | 49 | 46 | 20 |
|  | 24 | | PX |  | Pingxiang | 22.1 | 106.75 | 2014 | | 50 | | 48 | 50 | 20 |
|  | 25 | | GZ | Guangdong | Guangzhou | 23.158 | 113.351 | 2013 | | 50 | | 48 | 40 | 20 |
|  | 26 | | MZ |  | Meizhou | 24.289 | 116.122 | 2005 | | 50 | | 46 | 47 | 20 |
|  | 27 | | SG |  | Shaoguan | 24.81 | 113.598 | 2015 | | 49 | | 42 | 39 | 20 |
|  | 28 | | ZQ |  | Zhaoqing | 23.047 | 112.465 | 2007 | | 50 | | 37 | 30 | 20 |
|  | 29 | | FZ | Fujian | Fuzhou | 26.083 | 119.234 | 2014 | | 50 | | 49 | 50 | 19 |
|  | 30 | | NP |  | Nanping | 26.581 | 118.095 | 2010 | | 15 | | 15 | 14 | 9 |
|  | 31 | | XM |  | Xiamen | 24.448 | 118.062 | 2010 | | 36 | | 41 | 40 | 20 |
|  | 32 | | XG | Jiangxi | Xingguo | 26.335 | 115.348 | 2014 | | 50 | | 48 | 50 | 20 |
|  | 33 | | JY | Hunan | Jiangyong | 25.274 | 111.344 | 2014 | | 48 | | 29 | 39 | 20 |
|  | 34 | | GZXY | Guizhou | Xingyi | 25.092 | 104.895 | 2014 | | 49 | | 49 | 48 | 20 |
|  | 35 | | PZH | Sichuan | Panzihua | 26.421 | 101.762 | 2014 | | 50 | | 50 | 50 | 20 |
|  | 36 | | VN | Vietnam | Hanoi | 21.033 | 105.817 | 2014 | | 50 | | 48 | 40 | 20 |
|  | 37 | | LB | Laos | Louangphabang | 20.1 | 102.583 | 2008 | | 47 | | 47 | 46 | 20 |
|  | 38 | | VT |  | Vientiane | 17.967 | 102.6 | 2008 | | 39 | | 39 | 31 | 20 |
|  | 39 | | ST | Thailand | Sisaket | 15.017 | 104.25 | 2009 | | 43 | | 36 | 33 | 20 |
|  | 40 | | PT |  | Pathum Thani | 14.017 | 100.733 | 2011 | | 49 | | 48 | 42 | 20 |
|  | 41 | | CM |  | Chiang Mai | 18.733 | 98.917 | 2010 | | 50 | | 42 | 35 | 20 |
|  | 42 | | MM | Myanmar | Mandalay | 21.983 | 96.05 | 2005 | | 46 | | 43 | 46 | 20 |
| **Southern SE Asia** | 43 | | MY | Malaysia |  | 3.022 | 101.706 | 2015 | | 50 | | 48 | 45 | 10 |
|  | 44 | | ID | Indonesia | Majalengka | -6.853 | 108.226 | 2015 | | 50 | | 45 | 33 | 20 |
|  | 45 | | PHD | Philippines | Dumaguete | 9.307 | 123.305 | 2015 | | 50 | | 49 | 48 | 20 |
|  | 46 | | PHL |  | Legazpi | 13.139 | 123.744 | 2015 | | 29 | | 28 | 25 | 20 |
|  | 47 | | PNG | Papua New Guinea |  | -6.315 | 143.956 | 2015 | | 40 | | 33 | 40 | 20 |
| **South**  **Asia** | 48 | | BD | Bangladesh | Dhaka | 23.98 | 90.28 | 2014 | | 50 | | 50 | 49 | 20 |
|  | 49 | | INBP | India | Patna | 25.705 | 84.975 | 2009 | | 50 | | 46 | 48 | 8 |
|  | 50 | | INHS |  | Solan | 30.92 | 77.12 | 2009 | | 28 | | 28 | 26 | 19 |
|  | 51 | | INMM |  | Mumbai | 19.076 | 72.878 | 2009 | | 50 | | 32 | 36 | 20 |
| **Africa** | 52 | | SE | Senegal |  | 14.497 | -14.452 | 2015 | | 50 | | 13 | 29 | 20 |
|  | 53 | | ML | Mali |  | 12.613 | -8.015 | 2015 | | 43 | | 16 | 16 | 20 |
|  | 54 | | GN | Guinea |  | 9.946 | -9.697 | 2015 | | 50 | | 19 | 22 | 20 |
|  | 55 | | KT | Cote d'Ivoire |  | 7.54 | -5.547 | 2015 | | 50 | | 41 | 34 | 20 |
|  | 56 | | BF | Burkina Faso |  | 12.238 | -1.562 | 2015 | | 50 | | 42 | 45 | 20 |
|  | 57 | | BJ | Benin |  | 9.308 | 2.316 | 2015 | | 50 | | 21 | 32 | 20 |
|  | 58 | | NG | Nigeria |  | 9.082 | 8.675 | 2015 | | 20 | | 10 | 15 | 17 |
|  | 59 | | KE | Kenya |  | -1.29 | 37.415 | 2015 | | 50 | | 48 | 44 | 18 |
|  | 60 | | BI | Burundi |  | -3.373 | 29.919 | 2009 | | 50 | | 43 | 50 | 20 |
|  | 61 | | ZALT | South Africa | Tzaneen | -23.831 | 30.135 | 2015 | | 50 | | 42 | 49 | 20 |
|  | 62 | | KZN |  |  | -28.891 | 31.903 | 2015 | | 50 | | 42 | 49 | 20 |
| **Hawaii** | 63 | | HI | the US | Hawaii | 21.311 | -157.811 | 2015 | | 50 | | 38 | 50 | 20 |
|  |  | | **Total** |  |  |  |  |  | | **2867** | | **2442** | **2426** | **1216** |

Table S2 Primer sequences used in this study

| Gene | Name | Primers (5`-3`) | Tm（℃） | size (bp) |
| --- | --- | --- | --- | --- |
| *cox1*-a | dorF1507-1527  dorR2414-2436 | GTCTATCGCCTAAACTTCAGC  GCACGAGTATCTACATCTATTCC | 50 | 930 |
| *cox1*-b | dorF2333-2355  dorR3079-3101 | GGATCTCTAGGAATAATTTATGC  TTAAATCCATTGCACTAATCTGC | 50 | 770 |
| *nad6* | ND6 F  ND6 R | CATTGGTCTTGTAAAC  CATCCATAATTTACATCACG | 55 | 869 |

Table S3 Genetic variability in 63 populations of *B. dorsalis* based on microsatellite data and sequences

|  |  | SSR |  |  |  |  |  |  |  | *cox1* |  |  |  |  | *nad6* |  |  |  |  |
| --- | --- | --- | --- | --- | --- | --- | --- | --- | --- | --- | --- | --- | --- | --- | --- | --- | --- | --- | --- |
|  | Code | Size | *N*_A_ | *N*_E_ | *H*_O_ | *H*_E_ | *A*_R_ | *A*_N_ | *H*_S_ | size | N | *Hd* | $\pi$ | *k* | size | N | *Hd* | $\pi$ | *k* |
| 1 | ZJ | 50 | 7 | 2.607 | 0.437 | 0.554 | 4.945 | 0.08 | 0.555 | 43 | 34 | 0.984 | 0.00583 | 8.673 | 50 | 29 | 0.972 | 0.00764 | 4.009 |
| 2 | YX | 50 | 8.182 | 3.156 | 0.478 | 0.63 | 5.697 | 0.092 | 0.632 | 26 | 20 | 0.972 | 0.00604 | 8.982 | 39 | 25 | 0.947 | 0.00687 | 3.606 |
| 3 | SHBS | 50 | 7.546 | 3.369 | 0.49 | 0.655 | 5.475 | 0.097 | 0.657 | 30 | 17 | 0.926 | 0.00643 | 9.566 | 47 | 15 | 0.858 | 0.00845 | 4.437 |
| 4 | FY | 39 | 6.727 | 2.807 | 0.459 | 0.599 | 5.194 | 0.085 | 0.601 | 39 | 19 | 0.914 | 0.00502 | 7.463 | 39 | 17 | 0.88 | 0.00672 | 3.528 |
| 5 | HF | 50 | 6.909 | 2.91 | 0.438 | 0.596 | 5.054 | 0.1 | 0.598 | 49 | 22 | 0.935 | 0.00673 | 10.011 | 10 | 7 | 0.911 | 0.01101 | 5.778 |
| 6 | WH | 41 | 7 | 3.357 | 0.459 | 0.635 | 5.334 | 0.101 | 0.637 | 41 | 17 | 0.898 | 0.00455 | 6.763 | 43 | 13 | 0.88 | 0.00707 | 3.71 |
| 7 | CS | 49 | 8 | 2.695 | 0.487 | 0.581 | 5.494 | 0.065 | 0.582 | 42 | 35 | 0.988 | 0.00671 | 9.988 | 48 | 32 | 0.952 | 0.00771 | 4.05 |
| 8 | CQ | 50 | 8.273 | 3.449 | 0.458 | 0.651 | 5.718 | 0.122 | 0.653 | 48 | 37 | 0.983 | 0.00692 | 10.3 | 49 | 32 | 0.967 | 0.00765 | 4.017 |
| 9 | TW | 41 | 7.182 | 3.214 | 0.492 | 0.636 | 5.375 | 0.093 | 0.638 | 34 | 30 | 0.991 | 0.00696 | 10.349 | 39 | 21 | 0.945 | 0.00611 | 3.209 |
| 10 | DZ | 50 | 7.909 | 3.439 | 0.501 | 0.643 | 5.72 | 0.085 | 0.645 | 20 | 19 | 0.995 | 0.00792 | 11.784 | 19 | 11 | 0.912 | 0.00706 | 3.708 |
| 11 | HK | 50 | 8.091 | 3.108 | 0.508 | 0.624 | 5.564 | 0.075 | 0.625 | 48 | 47 | 0.999 | 0.00746 | 11.095 | 48 | 35 | 0.948 | 0.00678 | 3.561 |
| 12 | JFL | 30 | 6.909 | 3.102 | 0.461 | 0.602 | 5.458 | 0.08 | 0.605 | 48 | 42 | 0.993 | 0.00609 | 9.069 | 49 | 33 | 0.974 | 0.00703 | 3.692 |
| 13 | SY | 50 | 8.273 | 3.264 | 0.487 | 0.655 | 5.708 | 0.1 | 0.656 | 30 | 23 | 0.961 | 0.00665 | 9.894 | 50 | 27 | 0.94 | 0.00536 | 3.349 |
| 14 | WC | 20 | 6.455 | 3.587 | 0.533 | 0.65 | 5.743 | 0.07 | 0.654 | 20 | 19 | 0.995 | 0.00818 | 12.168 | 17 | 13 | 0.926 | 0.00756 | 3.971 |
| 15 | JS | 50 | 8.455 | 3.004 | 0.536 | 0.594 | 5.476 | 0.042 | 0.595 | 50 | 48 | 0.998 | 0.00696 | 10.362 | 28 | 21 | 0.963 | 0.00669 | 3.513 |
| 16 | JH | 44 | 9 | 3.414 | 0.471 | 0.643 | 5.923 | 0.098 | 0.645 | 43 | 40 | 0.997 | 0.00861 | 12.81 | 13 | 10 | 0.962 | 0.00781 | 4.103 |
| 17 | KM | 29 | 6.818 | 3.208 | 0.447 | 0.638 | 5.339 | 0.118 | 0.641 | 31 | 29 | 0.992 | 0.00806 | 11.989 | 21 | 18 | 0.971 | 0.00726 | 3.81 |
| 18 | QSH | 48 | 7.909 | 3.09 | 0.43 | 0.65 | 5.292 | 0.106 | 0.607 | 46 | 46 | 1 | 0.0088 | 13.088 | 50 | 38 | 0.953 | 0.00794 | 4.168 |
| 19 | RL | 45 | 7.909 | 2.92 | 0.505 | 0.592 | 5.389 | 0.049 | 0.593 | 45 | 45 | 1 | 0.00709 | 10.545 | 40 | 25 | 0.909 | 0.00612 | 3.214 |
| 20 | WS | 50 | 8.364 | 3.166 | 0.479 | 0.633 | 5.718 | 0.093 | 0.635 | 24 | 23 | 0.996 | 0.00654 | 9.736 | 29 | 18 | 0.933 | 0.00685 | 3.596 |
| 21 | BS | 50 | 7.818 | 3.321 | 0.484 | 0.643 | 5.407 | 0.097 | 0.645 | 47 | 40 | 0.984 | 0.00661 | 9.836 | 50 | 34 | 0.966 | 0.0075 | 3.94 |
| 22 | FC | 50 | 7.636 | 3.051 | 0.445 | 0.621 | 5.28 | 0.102 | 0.623 | 50 | 39 | 0.961 | 0.00584 | 8.696 | 47 | 26 | 0.94 | 0.00772 | 4.054 |
| 23 | NN | 50 | 7.727 | 3.208 | 0.471 | 0.631 | 5.381 | 0.091 | 0.633 | 49 | 38 | 0.981 | 0.00709 | 10.553 | 46 | 32 | 0.965 | 0.00719 | 3.777 |
| 24 | PX | 50 | 7.636 | 3.171 | 0.448 | 0.619 | 5.358 | 0.098 | 0.62 | 48 | 45 | 0.997 | 0.00602 | 9.857 | 50 | 39 | 0.985 | 0.00946 | 4.968 |
| 25 | GZ | 50 | 8.818 | 3.98 | 0.482 | 0.716 | 6.331 | 0.133 | 0.718 | 48 | 31 | 0.949 | 0.00598 | 8.897 | 40 | 22 | 0.938 | 0.00829 | 4.35 |
| 26 | MZ | 50 | 7.636 | 3.035 | 0.484 | 0.629 | 5.326 | 0.091 | 0.631 | 46 | 23 | 0.946 | 0.00648 | 9.648 | 47 | 15 | 0.876 | 0.00763 | 4.007 |
| 27 | SG | 49 | 7.364 | 3.085 | 0.502 | 0.635 | 5.311 | 0.077 | 0.637 | 42 | 27 | 0.957 | 0.00567 | 8.438 | 39 | 20 | 0.953 | 0.00853 | 4.478 |
| 28 | ZQ | 50 | 7.455 | 2.878 | 0.523 | 0.592 | 5.453 | 0.052 | 0.593 | 37 | 30 | 0.986 | 0.00758 | 11.282 | 30 | 24 | 0.984 | 0.01068 | 5.605 |
| 29 | FZ | 50 | 7.455 | 3.291 | 0.449 | 0.64 | 5.484 | 0.114 | 0.642 | 49 | 21 | 0.895 | 0.00686 | 10.207 | 50 | 13 | 0.838 | 0.00577 | 3.03 |
| 30 | NP | 15 | 5.364 | 3.417 | 0.436 | 0.665 | 5.182 | 0.133 | 0.673 | 15 | 10 | 0.993 | 0.00492 | 7.341 | 14 | 7 | 0.879 | 0.0055 | 2.89 |
| 31 | XM | 36 | 6.364 | 2.986 | 0.46 | 0.618 | 5.059 | 0.093 | 0.62 | 41 | 22 | 0.943 | 0.00655 | 9.751 | 40 | 22 | 0.935 | 0.00735 | 3.858 |
| 32 | XG | 50 | 7.182 | 2.837 | 0.479 | 0.604 | 5.342 | 0.076 | 0.605 | 48 | 17 | 0.883 | 0.00509 | 7.569 | 50 | 15 | 0.922 | 0.0067 | 3.515 |
| 33 | JY | 48 | 7.364 | 3.242 | 0.496 | 0.656 | 5.486 | 0.084 | 0.658 | 29 | 24 | 0.98 | 0.00544 | 8.809 | 39 | 27 | 0.947 | 0.00798 | 4.189 |
| 34 | GZXY | 49 | 9 | 3.303 | 0.504 | 0.66 | 6.09 | 0.096 | 0.662 | 49 | 32 | 0.956 | 0.00853 | 12.688 | 48 | 23 | 0.92 | 0.01039 | 5.456 |
| 35 | PZH | 50 | 7.546 | 3.408 | 0.49 | 0.62 | 5.322 | 0.071 | 0.621 | 50 | 25 | 0.943 | 0.00777 | 11.555 | 50 | 18 | 0.909 | 0.00791 | 4.153 |
| 36 | VN | 50 | 8.182 | 3.333 | 0.549 | 0.633 | 5.854 | 0.054 | 0.634 | 48 | 35 | 0.978 | 0.00664 | 9.876 | 40 | 24 | 0.909 | 0.00792 | 4.159 |
| 37 | LB | 47 | 8 | 3.557 | 0.395 | 0.647 | 5.562 | 0.144 | 0.65 | 47 | 43 | 0.996 | 0.00807 | 12.006 | 46 | 35 | 0.966 | 0.00929 | 4.878 |
| 38 | VT | 39 | 8.455 | 3.54 | 0.456 | 0.67 | 5.847 | 0.135 | 0.673 | 39 | 37 | 0.997 | 0.0091 | 13.544 | 31 | 26 | 0.968 | 0.00956 | 5.019 |
| 39 | ST | 43 | 8.455 | 3.351 | 0.491 | 0.609 | 5.886 | 0.079 | 0.611 | 36 | 33 | 0.995 | 0.00809 | 12.033 | 33 | 23 | 0.966 | 0.00922 | 4.841 |
| 40 | PT | 49 | 8.273 | 3.546 | 0.493 | 0.654 | 5.689 | 0.095 | 0.656 | 48 | 42 | 0.993 | 0.00716 | 10.66 | 42 | 34 | 0.987 | 0.00846 | 4.44 |
| 41 | CM | 50 | 8.546 | 3.191 | 0.53 | 0.63 | 5.625 | 0.055 | 0.631 | 42 | 41 | 0.999 | 0.00677 | 10.075 | 35 | 28 | 0.978 | 0.00849 | 4.457 |
| 42 | MM | 46 | 7.273 | 2.644 | 0.399 | 0.54 | 4.878 | 0.093 | 0.541 | 43 | 33 | 0.98 | 0.00602 | 8.959 | 46 | 32 | 0.967 | 0.0087 | 4.57 |
| 43 | MY | 50 | 7.727 | 2.835 | 0.475 | 0.603 | 5.182 | 0.086 | 0.605 | 48 | 38 | 0.986 | 0.00788 | 11.729 | 45 | 29 | 0.97 | 0.00973 | 5.109 |
| 44 | ID | 50 | 7.636 | 2.882 | 0.425 | 0.602 | 5.078 | 0.106 | 0.603 | 45 | 32 | 0.968 | 0.00871 | 12.958 | 33 | 13 | 0.67 | 0.00867 | 4.553 |
| 45 | PHD | 50 | 6.818 | 3.017 | 0.506 | 0.625 | 4.939 | 0.069 | 0.626 | 49 | 26 | 0.929 | 0.01017 | 15.129 | 48 | 12 | 0.838 | 0.00861 | 4.522 |
| 46 | PHL | 29 | 5.455 | 3.203 | 0.467 | 0.643 | 4.721 | 0.092 | 0.646 | 28 | 23 | 0.976 | 0.01365 | 20.315 | 25 | 13 | 0.897 | 0.01271 | 6.673 |
| 47 | PNG | 40 | 3.818 | 1.92 | 0.325 | 0.428 | 3.09 | 0.092 | 0.429 | 33 | 5 | 0.57 | 0.004 | 5.947 | 40 | 2 | 0.05 | 0.00124 | 0.65 |
| 48 | BD | 50 | 7.182 | 2.931 | 0.513 | 0.579 | 5.146 | 0.049 | 0.58 | 50 | 42 | 0.993 | 0.00664 | 9.88 | 49 | 36 | 0.975 | 0.00757 | 3.973 |
| 49 | INBP | 50 | 7.727 | 3.317 | 0.526 | 0.618 | 5.544 | 0.053 | 0.619 | 46 | 33 | 0.977 | 0.00719 | 10.7 | 48 | 25 | 0.931 | 0.00709 | 3.772 |
| 50 | INHS | 28 | 6.909 | 3.328 | 0.5 | 0.622 | 5.63 | 0.08 | 0.624 | 28 | 24 | 0.987 | 0.00812 | 12.085 | 26 | 16 | 0.883 | 0.00804 | 4.222 |
| 51 | INMM | 50 | 7.455 | 3.06 | 0.488 | 0.598 | 5.402 | 0.072 | 0.599 | 32 | 26 | 0.986 | 0.00799 | 11.889 | 36 | 19 | 0.881 | 0.00733 | 3.848 |
| 52 | SE | 50 | 4.909 | 2.567 | 0.421 | 0.535 | 4.003 | 0.09 | 0.536 | 13 | 4 | 0.423 | 0.00331 | 4.923 | 29 | 4 | 0.574 | 0.00346 | 1.818 |
| 53 | ML | 43 | 4.636 | 2.475 | 0.473 | 0.557 | 3.797 | 0.091 | 0.558 | 16 | 3 | 0.542 | 0.00412 | 6.125 | 16 | 3 | 0.575 | 0.0031 | 1.625 |
| 54 | GN | 50 | 4.818 | 2.768 | 0.457 | 0.577 | 4.065 | 0.059 | 0.579 | 19 | 8 | 0.766 | 0.00392 | 5.836 | 22 | 3 | 0.515 | 0.00294 | 1.545 |
| 55 | KT | 50 | 5.182 | 2.557 | 0.456 | 0.528 | 3.911 | 0.158 | 0.528 | 41 | 10 | 0.663 | 0.00429 | 6.383 | 34 | 6 | 0.597 | 0.00377 | 1.977 |
| 56 | BF | 50 | 4.273 | 2.51 | 0.409 | 0.52 | 3.619 | 0.057 | 0.521 | 42 | 5 | 0.409 | 0.00293 | 4.36 | 45 | 3 | 0.402 | 0.0024 | 1.261 |
| 57 | BJ | 50 | 6.364 | 2.795 | 0.317 | 0.538 | 4.636 | 0.044 | 0.541 | 21 | 6 | 0.695 | 0.00375 | 5.581 | 32 | 5 | 0.51 | 0.00279 | 1.464 |
| 58 | NG | 20 | 4.091 | 2.331 | 0.404 | 0.499 | 3.877 | 0.048 | 0.502 | 10 | 5 | 0.844 | 0.00439 | 6.533 | 15 | 2 | 0.527 | 0.00301 | 1.582 |
| 59 | KE | 50 | 3.364 | 2.172 | 0.38 | 0.454 | 3.025 | 0.076 | 0.455 | 48 | 5 | 0.41 | 0.00299 | 4.455 | 44 | 4 | 0.489 | 0.00239 | 1.254 |
| 60 | BI | 50 | 3.636 | 2.424 | 0.452 | 0.518 | 3.252 | 0.057 | 0.519 | 43 | 8 | 0.65 | 0.00456 | 6.78 | 50 | 3 | 0.513 | 0.00292 | 1.531 |
| 61 | ZALT | 50 | 3.818 | 2.307 | 0.425 | 0.506 | 3.216 | 0.099 | 0.507 | 42 | 7 | 0.617 | 0.00431 | 6.41 | 49 | 3 | 0.523 | 0.00293 | 1.541 |
| 62 | KZN | 50 | 4 | 2.431 | 0.452 | 0.518 | 3.377 | 0.057 | 0.518 | 42 | 11 | 0.549 | 0.00305 | 4.537 | 49 | 4 | 0.346 | 0.00222 | 1.163 |
| 63 | HI | 50 | 3.182 | 2.298 | 0.313 | 0.412 | 2.82 | 0.083 | 0.413 | 38 | 8 | 0.636 | 0.00148 | 2.209 | 50 | 3 | 0.496 | 0.00187 | 0.98 |

*N*_A_: mean number of alleles; *N*_E_: mean number of effective alleles; *H*_O_: mean observed heterozygosity; *H*_E_: mean expected heterozygosity; *A*_R_: mean allelic richness; *A*_N_: mean frequency of null alleles; *H*_S_: gene diversity. N: number of haplotypes in each population; *Hd*: haplotype diversity;$\pi$: nucleotide diversity; *k*: average numbers of nucleotide. differences.

Table S4 Pairwise *F*_ST_ among 63 populations of *B. dorsalis* based on microsatellite data

Values in bold are significant at P<0.05; values above 0.100 are indicated.

Table S5 Pairwise *F*_ST_ among 63 populations of *B. dorsalis* based on *cox1* data (below diagonal) and *nad6* data (above diagonal)

Values in bold are significant at P<0.05; values above 0.100 are indicated.

Table S6 Demographic history parameters among six groups of *B. dorsalis* based on sequences data

|  | Code | *cox1* | | | | | | *nad6* | | | | | |
| --- | --- | --- | --- | --- | --- | --- | --- | --- | --- | --- | --- | --- | --- |
|  |  | *Ɵ*_0_ | *Ɵ*_1_ | T | Tajima’s *D* | Fu’s *F*_s_ | SSD | *Ɵ*_0_ | *Ɵ*_1_ | T | Tajima’s *D* | Fu’s *F*_s_ | SSD |
| 1 | CC | 1.559 | 106.445 | 8.3 | **-2.281** | **-24.078** | 0.0008 | 0.012 | 31.786 | 4.498 | **-2.288** | **-25.291** | 0.0007 |
| 2 | SCNA | 2.742 | 321.875 | 8.0 | **-2.411** | **-23.502** | 0.0001 | 0.007 | 54.431 | 4.381 | **-2.415** | **-24.724** | 0.0005 |
| 3 | SSA | 7.552 | 47.695 | 6.7 | **-1.837** | **-23.811** | 0.006 | 2.384 | 12.118 | 5.234 | **-1.729** | **-24.923** | 0.0147 |
| 4 | SA | 0 | 221.563 | 8.6 | **-2.075** | **-24.190** | **0.019** | 0.056 | 18.033 | 4.426 | **-2.290** | **-25.742** | 0.0022 |
| 5 | AF | 0 | 99999 | 0 | -0.776 | -9.654 | **0.605** | 0.000 | 2.317 | 3.961 | -1.207 | **-0.864** | **0.130** |
| 6 | HI | 0.002 | 2.710 | 5.393 | 0.092 | -0.519 | 0.105 | 0.000 | 1.367 | 2.715 | 0.958 | 2.036 | **0.156** |

*Ɵ***_0_**: effective populations sizes before expansion; *Ɵ*_1_: effective populations sizes after expansion; T: time of population expansion; SSD: sum of square deviation between expected and observed mismatch distribution under the sudden expansion model. Bold values are significant at P<0.05.
